# Editorial

**Published:** 1867-09

**Authors:** 


### Editorial.

DUFFIELD, PARKE & CO.—On the last page of the cover will be found the advertisment of the above Drug and Chemical Manufacturing Company of Detroit, Michigan. We think it is one of the best and most reliable establishments in our country.

CHICAGO MEDICAL SOCIETY.—At the regular meeting of the Chicago Medical Society, held on the evening of August 23d, 1867, Dr. R. M. Lackey presented a report concerning the sanitary condition of the West Division of the city, and the prevalence of diseases. The report presented several items of interest, and led to a discussion, in which several members participated.

Dr. Fenn, resident-physician, or interne, of the County Hospital, presented two pathological specimens, obtained from *post mortems* at the Hospital. The first, was the kidneys from a young woman, who died from pulmonary phthisis. For several weeks before her death the stomach had tolerated but little food, and rejected cod-liver oil. Her lower extremities had been œdematous; the urine scanty, and highly albuminous. The kidneys, as presented to the society, were about normal size. On peeling off the capsule, or external membrane, several small tubercular deposits were found between it and the kidney; the substance of the latter was somewhat softened, and the cortical part extensively changed by fatty degeneration. Several of the pyramidal bodies were so completely transformed into fatty tissue as to have lost their natural red and striated appearance, and a large part of the cortical substance had undergone the same change.

The second, was a portion of the sigmoid flexure of the colon, from a boy who had died with dysentery. The boy had been in this country only a few weeks, and died after an illness of about ten days. His symptoms were those common to dysentery, accompanied by a strongly-marked typhoid condition of the system. In the section of the intestine presented, the mucous membrane was intensely red, with spots of dark brown; much tumefied, partly by infiltration into the sub-mucous cellular tissue, and distinctly ulcerated. The same changes were found to exist throughout the whole extent of the colon and rectum.

Dr. A. Fisher announced to the Society the death of Dr. Orrin Smith, one of its oldest and most respected members, and moved the appointment of a committee to report resolutions expressive of the sentiments of the Society. The motion was adopted, and the Chair appointed Drs. Fisher, Davis, Paoli, Hamill, and Wickersham as such committee.

Drs. Fisher and Ross stated to the Society the results of the *post mortem* examination of Dr. Smith. He had been in feeble health for many years, but continued in active practice until within a few weeks of his death. The pericardium was found closely adherent to the heart, throughout its whole extent; the heart much enlarged, with softening of its structure and dilatation of its cavities; the semilunar valves of the aorta and the mitral valve much thickened and of cartilaginous hardness; extensive pleuritic adhesions in the left side of the chest, and partial hepatization of the lower lobe of the lung. No morbid changes in other organs were noted. The pleuritic and pericardial adhesions had evidently existed many years.

Dr. Fisher, from the committee, presented the following preamble and resolutions, which were unanimously adopted:

*Whereas,* It has pleased the Supreme Ruler to remove from our midst, and take to Himself a prominent member of this Society, by the death of Dr. Orrin Smith; therefore, as a tribute of respect, we offer the following resolutions:

*Resolved,* That, in the death of Dr. Smith, this Society has been deprived of an honorable member, the community of an experienced practitioner and a valued citizen.

*Resolved,* That we recognize in our departed friend and brother, a man of great energy and perseverance, so entirely devoted to his profession and to the cause of humanity that, although advanced in life, and for many years a sufferer from organic disease of the heart, he continued to practice his profession till a few weeks before his death.

*Resolved,* That we tender to the afflicted family of the deceased our earnest sympathy in their sad bereavement and irreparable loss.

*Resolved,* That the Secretary of this Society furnish a copy of these resolutions to the family of the deceased, to each of the daily papers of the city, to the *Chicago Medical Journal,* and the MEDICAL EXAMINER.

New Medical Journals.—We have received the first number of the *Leaveaworth Medical Herald*, edited by C. A. Logan, M.D., and T. Links, M.D., and published at Leavenworth, Kansas. It presents every evidence of being worthy of the patronage of the profession. The *Western Journal of Medicine and Surgery*, published at Indianapolis, also comes regularly, filled with interesting matter.

---

Medical Education in Chicago.—For many years a considerable number of Medical students residing in the North-western States, have resorted to the Medical Colleges in New York and Philadelphia, almost wholly on account of the supposed superior advantages for clinical study in the hospitals of these cities. However valid such an excuse may have been ten or fifteen years since, it is entirely groundless at the present time. In the present number of the Examiner, we publish the Clinical arrangements of the Cook County or Almshouse Hospital of this city, and those of the Chicago Eye and Ear Infirmary; and if we add to these the advantages which have long been afforded in the Mercy Hospital, directly under the charge of the Faculty of the Chicago Medical College; and of the St. Luke's Hospital, it will be seen that Chicago presents, to-day, as wide a field for clinical instruction and study, as can be occupied by any student or practitioner in the country.

Indeed, we doubt whether a more extensive and complete arrangement for instruction in all the departments of clinical study, can be found in any city in this country. The arrangement includes not only an ample field of practical surgery and practical medicine, but a special course in the Mercy Hospital on Diseases of the Heart and Respiratory organs, including physical diagnosis and the use of the Laryngoscope; special attention to *post mortems* and Pathological anatomy in the County hospital; and special courses of instruction on diseases of the Eye and Ear, by Dr. Hildreth, in the Eye wards of the County hospital, and by Dr. Holmes in the Eye and Ear Infirmary, including the use of the Opthalmoscope and all other modern appliances in diagnosis.

If we add to these ample facilities for clinical instruction and study, the full curriculum, the systematic order, and the lengthened lecture term of the Chicago Medical College, we shall have presented to the profession as complete an arrangement for affording a thorough education in all the departments of Medical science and practice, as can be found any where on this side of the Atlantic.

We cordially invite every student and practitioner in the country, to visit our Medical Institutions, and satisfy themselves concerning the correctness of our statements.

---

DEATHS.—The last number of the Boston *Medical and Surgical Journal*, contains an account of the death of John Mason Warren, of that city; and the daily papers yesterday, announced the death of James Jackson, of the same place, in the 89th year of his age. Thus have passed away two of the most distinguished members of our profession, members who have conferred honor upon the profession to which they belonged.

---

### COOK COUNTY HOSPITAL.

The Cook County Hospital, is in fact the almshouse hospital for this city and county. It fills the same place here that Bellevue does in New York.

Its managers have exhibited both wisdom and liberality, in opening its wards for systematic and efficient clinical instruction. Wisdom, because a public hospital constantly open for clinical instruction to students and practitioners, will always be more punctually and faithfully served by the attending physicians and surgeons, and the wants of the sick better cared for, than where no such instruction is given. Liberality, because, instead of making it the *attache* of any particular college, they have selected for the Medical and Surgical Board, some of the more active, enterprizing, and efficient members of the profession who are not connected with either of the Medical Colleges in the city, but who will teach all students who seek its advantages and comply with its regulations.

Dr. Edwin Powell, whose name appears in the list of attending surgeons, has resigned his chair in Rush Medical College, for the express purpose of accepting the place of surgeon to the Hospital. As at present organized, we do not hesitate to assure students throughout the North-West, that the Cook County Hospital in this city, affords them just as good facilities for the Clinical study of disease, including Morbid Anatomy, as can be found in Bellevue, New York, the Pennsylvania Hospital at Philadelphia, or the Chaity Hospital in New-Orleans. We take pleasure in appending the following official cicular issued by the Medical Board of the Hospital:—

Cook County Hospital, City of Chicago.—Winter Term of Clinical Instruction.—1867-68.—The Winter term of Clinical Instruction in the Cook County Hospital, will commence on the 1st of October, 1867, after the usual vacation of three months. The continued prosperity and growth of this Institution, with its large number of patients, representing every variety of disease, furnishes to the student an almost unlimited field for clinical study. The Annual Report for the year ending July 1st, 1867, shows that during the year *one thousand and thirty-seven* patients were treated in the Hospital. The number discharged during the year was *nine hundred and twenty*. During the year there were in the lying-in department *sixty-six* births. *Thirty-one* capital operations were performed in the surgical department. There were *seventy-three* autopsies, which afforded an excellent opportunity for the study of morbid anatomy. The field for the study of diseases of the chest is probably not excelled in any hospital in this country. In addition, the out-door, or Dispensary department of the Hospital, furnishes a large number of interesting cases. This is in charge of the medical staff of the Hospital, and is in operation from 1 to 2 o'clock P.M. every day of the week, (Sunday excepted,) affording an excellent clinic.

The Pathological Museum now contains many very interesting specimens, and is available for the study of morbid anatomy.

It is the purpose of the medical staff to use and develop the immense resources of the Hospital for the promotion of medical education, and to add whatever is possible to the means and facilities for medical study attainable in Chicago. The object for which they will earnestly labor being an elevated standard of professional knowledge among the students, and such junior practitioners as may seek our city for these objects.

There will be five clinics a week from October to July, and two a week during July, August and September. The clinical days are Tuesdays, Fridays and Saturdays, 1½ o'clock P.M. The medical clinics will be given by Drs. Ross, Bevan, Jones and Lyman; the surgical by Drs. Powell, Bogue, Smith and Fitch. Dr. Hildreth will hold an opthalmic clinic every Saturday.

Special courses of instruction will be given during the winter on Auscultation and Percussion, by Dr. Ross; on Microscopy and Morbid Anatomy by Dr. Lyman; on Surgical Dressings by Dr. Bogue; on the Opthalmoscope by Dr. Hildreth.

Fee for admission to the Hospital, $5.00. Graduate practitioners of medicine visiting the city, and desiring to follow the practice of the Hospital, are admitted without charge.

---

### MEDICAL ORGANIZATION IN CANADA.

---

The following indicates a movement in the right direction among our Canadian neighbors.—EDITOR.

REPORT.—*Whereas;* By the "British North American Act, 1867." the union of the Provinces of Canada, Nova Scotia and New Brunswick is effected, and united Legislative and Executive action is secured: and

*Whereas;* Closer connection must necessarily take place in all the relations of life; religious, moral, and social: and

*Whereas;* "Uniformity of Laws in Ontario, Nova Scotia, and New Brunswick" is provided for in the said Act: and

*Whereas;* Uniformity in the laws which regulate life and health, and especially those governing the exercise of the medical profession, stand preëminent:

*Therefore:* The Medical Society of Quebec,—the oldest city in the Dominion of Canada, deems it a duty to take action in the premises; and has come to the conclusion, that the most equitable, surest, and best means of attaining the desired end, will be by an union of the members of the medical profession of the Dominion of Canada in Conference, at as early a period as practicable after the consummation of the union of Canada takes place, under Her Most Gracious Majesty's proclamation.

*Wherefore:* The following resolutions were *unanimously* adopted, and are now respectfully submitted to the consideration of the medical profession of Canada, for such action as may be agreed upon in Conference.

*Resolved, 1.* That in the interest of the public, and the medical profession, it is desirable to adopt such means as will insure a *uniform* system of granting license to practice Medicine, Surgery, and Midwifery, throughout the Dominion of Canada.

*Resolved, 2.* That in future, all medical degrees or diplomas, of Universities, Colleges, or Schools of Medicine, shall have an honorary value, and licences to practice Medicine, Surgery or Midwifery, in the Dominion of Canada, shall be granted by a Central Board of Examiners, in each Province, before whom all holders of Degrees in Medicine, or Diplomas for Surgery, or Midwifery, shall appear for examination.

*Resolved, 3.* That a committee of seven members be named by the Medical Society, to confer with the various Universities, Colleges, and Medical Schools in Canada, on the subject of the establishment of a Central Board of Examiners, before which, all candidates for license to practice medicine in the Dominion of Canada, shall be examined.

*Resolved, 4.* That the Quebec Medical Society recommends the calling of a Convention of Medical Delegates, from Universities, Colleges, Schools, Medical Societies, etc., in the Dominion of Canada; to meet at the city of Quebec, on the second Wednesday in October, 1867, for the purpose of adopting some concerted action, on the subject of medical legislation, in conformity with this report, and for the formation of a "*Canadian Medical Society.*"

The whole respectfully submitted.

*Laval University. Quebec, 18th June, 1867.*

W. MAXWELL, M.D., *Chairman.*

R. H. RUSSELL, M.D., *Secretary.*

---

### NEW SYDENHAM SOCIETY.

---

We call special attention to the following notice of this Society.

1116 Girard St., PHILADELPHIA.

*Dear Sir,*—As Honorary Secretary of the New Sydenham Society, I beg to bring its publications under your notice, and to invite you to enrol yourself as a member.

The Society was instituted for the purpose of supplying certain acknowledged deficiencies in the existing means of diffusing medical literature. Works of a *practical* character and of *permanent* value are selected for publication.

You can acquire the whole of the works issued by the Society from 1859 to 1867 (35 volumes, including 28 vols. handsomely bound in cloth, gilt, and 7 fasciculi of the Society's Atlas of Portraits of Skin Diseases, embracing more than 20 life-size colored plates, 18×24 inches), for Nine Guineas; or you can commence with either the past or the present year.

The subscription is One Guinea per annum, payable in advance.

I undertake to receive your annual subscription, and to hand you the books as soon as they are issued.

I am, Dear Sir, truly Yours,
RICHARD J. DUNGLISON, M.D.,
*Honorary Local Secretary.*

*Series for 1866.*

I. Bernutz and Goupil on Diseases of Women. Vol. I; II. Fasciculus of Atlas of Portraits of Diseases of the Skin (three beautiful colored plates life size); III. Hebra on Diseases of the Skin. Vol. I; IV. Bernutz and Goupil on Diseases of Women. Vol. II.

*Series for 1867.*

I. Griesinger on Mental Diseases; II. Biennial Retrospect of Medicine and Surgery; III. Fasciculus of Atlas of Portraits of Disease of the Skin (colored plates); IV. Hebra on Diseases of the Skin. Vol. II.

Annual Subscription $7.50, in advance, (the duty, etc. payable on arrival of the vols., amounting to about $2.50 additional).

---

OBITUARY.—DEATH OF DR. ORREN SMITH.—Another of our most useful and valuable citizens has departed this life. With deep sorrow, which will find an echo in the heart of every man who knew him, we announce the death of Dr. Orren Smith, which took place at his residence, No. 236 Illinois Street, on Monday evening.

For years past the deceased had been troubled with heart disease. During all this time his life hung by a single thread, but by sheer force of will and tenacity of purpose, he clung to that life, which has been a real and substantial benefit to his fellow beings.

It is only recently, and at the earnest solicitations of his friends, that he gave up an extensive and lucrative practice, to become himself a patient, and receive the kind offices from others which he himself had so often extended to suffering humanity.

Dr. Smith was born in Washington County, Vermont, in July, 1806, and was consequently at the time of his death, sixty-one years of age. He graduated in 1830, as a medical student, at the University of Vermont, and practiced for upwards of twenty-five years at Montpelier and vicinity, where he was distinguished for his skill as a physician, and whence he was appointed in 1850 to the Professorship of Obstetrics and Diseases of women and children, in the University where he graduated, which position he filled with distinguished ability until 1857, when he resigned on account of ill-health, and left Vermont for a change of climate. After spending a year or two at the South, Dr. Smith finally, in December, 1857, settled in Chicago, where he continued in the practice of his profession up to the very hour of his last prostration and sickness.

---

New York Medical Socety on Consanguineous Marriages.—At the late meeting of the "Medical Society of the State of New York," it was resolved: "That a Committee be appointed to investigate and report upon the result of consanguineous marriages, etc."

If such marriages come under your observation, you will confer a favor by answering the following questions, and transmitting such report, before November next, to the undersigned, one of the Committee appointed:

1. Name (initials) and age of *Husband.*
2. Nativity.
3. Age when married.
4. Constitution.
5. Health, deformities, peculiar diathesis.
6. Health of his family, hereditary diseases, deformities, etc.
7. Name (initials) and age of *Wife.*
8. Nativity.
9. Age when married.
10. Constitution.
11. Health, deformities, peculiar diathesis.
12. Health of her family, hereditary diseases, deformities, etc.
13. How are the parties related to each other?
14. How long married?
15. How many children, or sterility.
16. Abortions; cause; how many, and at what period?
17. Children died, at what ages and from what diseases?
18. The constitution, age and present health of living children, deformities, mental conditions, idiocy, cretinism, deaf, mute, blind, epilepsy, albinism, insane, etc.

19. Remarks and other information.

Hoping to receive your valuable coöperation for the advancement of medical science,

I remain yours, most respectfully,

ROBERT NEWMAN, M.D.

*118 W. Houston St., New York, July, 1867.*

---

Clinical Instruction at the Chicago Charitable Eye and Ear Infirmary.—During the winter courses of instruction at the medical colleges of Chicago, there will be regular clinical lectures on diseases of the eye at the Infirmary. This institution offers the medical student and practicing physician unusual opportunities for the practical study of diseases of the eye and of their medical and surgical treatment.

During the past year, *five hundred and fifty-five* patients were treated at the Infirmary, of which *eighty-seven* required important surgical operations. An aggregate of *3197* patients have received the benefits of the institution since its organization, nearly ten years since.

There has been an average daily attendance of 26 patients at the infirmary during the past three months.

Dr. Holmes will give special courses on the use of the ophthalmoscope.

The Legislature of the State of Illinois, at its last regular session, appropriated the sum of $10,000, for the support of the poor of the State, during treatment, at the Infirmary for diseases of the eye or ear.

The Legislature of the State of Wisconsin recently appropriated the sum of $500, for the support of Wisconsin soldiers receiving treatment at the Infirmary for diseases of the eye, contracted in the army during the late war.

*Trustees*—W. L. Newberry, President; P. Carpenter, Vice-President; S. Stone, Secretary; E. B. McCagg, Treasurer; W. H. Brown, William Barry, T. B. Bryan, C. G. Hammond, E. C. Larned, Wesley Munger, E. W. Blatchford, Daniel Goodwin, Jr.

*Consulting Surgeons*—Prof. J. W. Freer, M.D., Prof. H. A. Johnson, M.D.

*Attending Surgeons*—E. L. Holmes, M.D., Prof. E. Powell, M.D.

*Superintendent*—G. Davenport. *Matron*—Mrs. Davenport.

### CAUSES OF DEATH.

| | | | |
|---|---|---|---|
| Accidents, | 16 | Fracture, Compound, (leg,) | 1 |
| Anasarca, | 1 | Gastritis, | 3 |
| Apoplexy, | 2 | Gastro Enteritis, | 1 |
| " Cerebral, | 2 | Gangrenous Sore-throat, | 1 |
| Brain Disease, | 1 | Hydrocephalus, | 17 |
| " Congestion of, | 6 | Heart Disease, | 2 |
| " Softening of, | 2 | Heart Disease, Valvular of, | 2 |
| " Concussion of, | 2 | Inflammation of Bowels, | 5 |
| " Inflammation of, | 4 | Inflammation of Liver, | 1 |
| Bright's Disease, | 1 | Inanition, | 4 |
| Bronchitis, | 3 | Jaundice, | 1 |
| Cancer Duodenum, | 2 | Lungs Atelectasis, | 1 |
| Canker, | 1 | Laryngitis, | 1 |
| Caries of Spine, | 1 | Measles, | 8 |
| Congestion of Lungs, | 3 | Meningitis, | 5 |
| Cholera, | 3 | Meningitis, Cerebro-Spinal, | 3 |
| Cholera-Morbus, | 3 | Mortification, | 1 |
| Cholera Infantum, | 177 | Old Age, | 2 |
| Cirrhosis of Liver, | 1 | Obstruction of Bowels, | 1 |
| Convulsions, | 33 | Over-exertion, | 1 |
| Cyanosis, | 1 | Paraplegia, | 1 |
| Disease of Chest, | 1 | Paralysis, | 3 |
| Diabetes, | 1 | Peritonitis, | 4 |
| Diarrhœa, | 25 | Pneumonia, | 10 |
| Debility, | 7 | Phthisis Pulmonalis, | 29 |
| Diphtheria, | 2 | Premature Birth, | 13 |
| Dysentery, | 10 | Breast, | 1 |
| Delirium Tremens, | 1 | Scirrhus Uterus, | 1 |
| Dyspepsia, | 2 | Small-Pox, | 6 |
| Dropsy Abdominal, | 1 | Stillborn, | 12 |
| Dropsy of Brain, | 1 | Stricture Intestines, | 1 |
| Encephalis, | 1 | Suicide, | 2 |
| Enteritis, | 6 | Sunstroke, | 1 |
| Exhaustion, | 1 | Suffocation, | 1 |
| Erysipelas, | 1 | Tabes Mesenterica, | 25 |
| Fever, Puerperal, | 2 | Teething, | 13 |
| Fever, Typhoid, | 9 | Tumor of Womb, | 1 |
| Fever, Typhus, | 2 | Whooping-Cough, | 4 |
| Fever, Scarlet, | 1 | Worms, | 1 |
| Fever, Intermittent, | 1 | Ulceration of Stomach, | 2 |
| Fungus Hæmatodes, | 1 | Unknown, | 7 |
| Total, | | | 538 |

### NATIVITIES.

| | | | | | |
|---|---|---|---|---|---|
| Chicago, | 242 | England, | 10 | On Sea, | 1 |
| Illinois, | 7 | Norway, | 9 | Switzerland, | 1 |
| Other parts U. S., | 84 | Sweden, | 12 | St. Helena, | 1 |
| Germany, | 60 | Shetland Isles, | 1 | France, | 1 |
| Ireland, | 35 | Prussia, | 3 | Darnstadt, | 1 |
| Bohemia, | 10 | Scotland, | 4 | Unknown, | 46 |
| Austria, | 2 | Canada, | 5 | | |
| Holland, | 2 | Denmark, | 1 | Total, | 538 |

AGES OF THE DECEASED.—Under 5 years, 363; over 5 and under 10 years, 17; over 10 and under 20, 10; over 20 and under 30, 36; over 30 and under 40, 29; over 40 and under 50, 19; over 50 and under 60, 6; over 60 and under 70, 7: over 70 and under 80, 4; over 80 and under 90, 2; still born, 16; unknown, 29. Total, 538.

MONEY RECEIPTS FROM JULY 26TH, TO AUGUST 30TH.—Drs. J. J. Fyke, Odin, Ill., $3; William B. Hart, Greenwood, Ill., 3; W. J. Johnson. Chicago, Ill., 1.50; J. W. Ghrist, Ackley, Ill., 3; J. Y. Campbell, Paxton, Ill., 3; W. M. Burbank, Barrington, Ill., 3; J. H. & D. McDill, Biggsville, Ill., 3; Wm. Martin, Chicago, Ill., 2.

---

TO PHYSICIANS.—By request, Prof. Horatio R. Storer will deliver his second private course of twelve Lectures upon the TREATMENT OF THE SURGICAL DISEASES OF WOMEN, during the first fortnight of December, at his rooms in Boston. Fee $50, and Diploma required to be shown.

Certificates of attendance upon the course just completed have been issued to the following gentlemen: Dr. C. M. Carleton, Norwich, Ct.; Daniel Mann, Pelham, N.H.; G. E. Bullard, Blackstone, Mass.; J. A. McDonough, Boston, Mass.; M. C. Talbott, Warren, Pa.; H. Gerould, Erie, Pa.; E. F. Upham, West Randolph, Vt.; W. L. Wells, Howall, Mich.; and W. A. I. Case, Hamilton, C.W.

*Hotel Pelham, Boston, 1st July, 1867.*

